# Florence Nightingale and the Royal National Pension Fund for Nurses

**Published:** 1916-07-01

**Authors:** 


### FLORENCE NIGHTINGALE

AND THE

### ROYAL NATIONAL PENSION FUND FOR NURSES.

### Letters and Correspondence of Historic Value.

AT the twenty-ninth annual general meeting of the Council of the Royal National Pension Fund for Nurses, on June 22, Mr. Thomas Charles Dewey, F.I.A., Deputy Chairman of the Pension Fund and Chairman of the Prudential Assurance Company, Ltd., read an interesting letter from Florence Nightingale, dated October 16, 1879, to Mr. A. W. Goodman, headed "Nurses' Provident Fund," a copy of which will be found below. The results of the inquiries then made bore no fruit. It will be noted that this letter to Miss Nightingale is dated 1879, an interesting fact, seeing that in 1878 the sufferings and misfortunes of Nurse Steer, who ultimately died in a workhouse, and her devotion to a Swedish sailor patient treated in that year at the Seamen's Hospital, Greenwich, as recorded by Dr. Potter in his "Story of the Royal National Pension Fund for Nurses," caused Mr. Henry C. Burdett, at that time Secretary-Superintendent of the Seamen's Hospital, Greenwich, to resolve that sick pay and pensions should be organised for all British nurses, and that steps must be taken and the necessary means provided to secure such a provision. Nurse Steer's case was not a solitary instance, but was the kind of thing which was constantly happening all over the country, and must recur with increasing frequency as hospitals and nurses were multiplied with the growth of the population.

This is not the place to attempt a history of the Pension Fund. It must suffice to say that from 1880 to 1883 Mr. Burdett devoted such leisure as he could command to preparing the ground for the establishment of a Nurses' Pension Fund. He continued these efforts until they eventuated in the large meeting referred to by Mr. Dewey in the year 1887. On February 16, 1888, the National Pension Fund was incorporated under the Life Insurance Companies Act and licensed by the Board of Trade under Section 23 of the Companies Act, 1867. On incorporation the Fund, from the business point of view, was stayed under the terms of No. 6 of the Articles of Association, which provided that "until the society shall have obtained proposals for the grant of 1,000 annuity policies no business shall be commenced, and if before the expiration of two years from January 1, 1888, proposals to that extent shall not have been received, the Society shall not undertake any business but shall be wound up." In other words, the four City merchants who advanced the £20,000 deposit required under the Insurance Acts to enable the Pension Fund to be established upon a sound financial basis accepted Sir Henry Burdett's suggestion as set out in Article 6, and threw the responsibility upon the whole of the nursing body to show their confidence and wish to obtain, mainly by their own thrift, an adequate and safe provision for their old age with sick pay during their working years.

It should now be noted that Sir Henry Burdett's letter to Miss Nightingale and her reply are dated 1889. This is important, because it affords striking evidence of how the two years of delay pending the commencement of business were occupied to secure so far as possible the co-operation of everybody of importance interested in nursing in this country. Miss Nightingale, in her letter, expresses deep interest in any scheme which tends to promote thrift among the nurses. She felt that the Pension Fund would undoubtedly be a great boon in the event of the nurses coming upon the Fund, but records her doubt that any but a small proportion of them would do so. Here we have Florence Nightingale's attitude clearly defined in regard to the National Pension Fund, with a further exhibition of her strong character in expressing her distaste for the tendency of the day to grant rewards and "special recognition" to individuals for something they may have done, and she deprecates the bringing of nurses' names before the public.

The following letters will excite universal interest. The first of them, as Sir Everard Hambro pointed out with prompt wisdom and appreciation, demonstrates the position occupied by the Prudential Assurance Co., Ltd., which almost from the outset has rendered material aid to the Council of the Pension Fund and to the nurses who have placed their savings in its custody. Mr. Thomas Charles Dewey, F.I.A., has rendered yeoman service to this Fund, and we hope that this and his other great services may without delay receive adequate State recognition.

The last two letters relate to the time when evidence was forthcoming that the nurses would take up one thousand annuity policies, an end accomplished before July 1, 1890. It had to be decided what should be done for the first thousand nurses who had displayed the courage and thrift to apply for them and had so secured the commencement of business by the National Pension Fund for Nurses and its initial success. This problem is explained in Mr. Burdett's letter, and Miss Florence Nightingale gives her opinion in her reply. Ultimately the four City merchants who, with Mr. Burdett, founded the Fund—namely, the late Lord Rothschild, the late Lord Aldenham, Sir Everard A. Hambro, K.C.V.O., and the late Mr. Junius S. Morgan, in making a gift of the original deposit of £20,000 to the Fund, specially allocated £5,000 of it to be invested at compound interest for the sole benefit of the first thousand nurses.

### FLORENCE NIGHTINGALE AND THE PENSION FUND—(*continued*).

### Florence Nightingale to Mr. A. W. Goodman.

Dear Sir,—I cannot thank you enough for your very kind letter of information and advice relating to the formation of a "Trained Nurses' Provident Fund," which if formed, we had some hopes might have been affiliated to your Prudential Assurance Company, and concerning which my cousin called at your offices . . . . Pray accept my most hearty thanks for your goodness in offering us your help in making inquiries, and even in contributing, if we were to take public subscriptions. I should feel very unwilling to do the latter if it were possible to float our Fund otherwise, because it would tend to encourage hospitals to lessen the pay which they ought to give to trained nurses, or not to raise it. The idea of this "Provident Fund" for Nurses is still quite in embryo. But I cannot help writing to you at once to thank you for your great kindness. And I shall certainly claim your promised help in advice and information, when we are a little more pledged, in knowing what our nurses can do. Pray believe me, my dear Sir, ever your faithful servant, (Signed) Florence Nightingale.

10 South Street, Park Lane, W., October 16, 1879.

### Mr. Burdett to Florence Nightingale.

Dear Miss Nightingale,—I have rejoiced to hear from time to time, from Mr. Bonham Carter, Miss Wardroper, and others, of your interest in the progress of the National Pension Fund for Nurses. I am sure you will be rejoiced to hear that so many nurses have now joined, and that the various hospitals and institutions are affiliating themselves to the Fund so rapidly that it may be regarded as an assured success, having taken its place amongst the permanent institutions of the country. In these circumstances, it is a matter for anxious consideration what shall be done for the nurses who, by coming spontaneously forward and entrusting their savings to the Fund—despite criticisms and carpings of various kinds—have materially contributed to this desired result. I have thought I might venture to consult you on this moot point, as I feel these pioneers ought to have some special recognition entailing substantial benefit. Apart from a special bonus of any kind, it is proposed that they should have an illuminated card, or token, or parchment, containing their individual names and stating how they enabled the Pension Fund to be permanently established. These proposals are not open to any of the objections which suggest themselves in other directions. If we were to give them a reception we should lay ourselves open to exactly the same criticisms as the Conversations of the British Nurses' Association, and we can, I feel, hardly keep ourselves too clear of the charge of seeking to bring forward nurses in the same sort of way. If it would save you any trouble, I would, of course, gladly come and talk the matter over quietly with you at any time.

In any case, I hope you will understand that, having recently gone over the various developments in hospital management throughout the civilised world, I have been struck with the evidence forthcoming of the enormous influence everywhere of your individual work and example. These created a revolution, and as I hope the National Pension Fund will revolutionise the lot of our nurses by relieving them of all anxiety for the future whilst teaching them thrift for the present. I feel it would not be right or just to take action at this juncture without learning your views on the points I have ventured to explain.

Hoping you are better in health, believe me, faithfully, yours, (Signed) Henry C. Burdett.

The Lodge, Porchester Square, June 4, 1889.

### Florence Nightingale to Mr. Burdett.

Dear Sir,—I beg to thank you for your kind note of June, and to hope that your kindness will excuse some delay on my part in acknowledging it. Under the severe pressure of business and illness, my ability is not equal to my will always to keep pace with my correspondence. With reference to the "National Pension Fund," I am deeply interested in any scheme which tends to promote thrift among the nurses. To those who join it and are able to keep up their payments it will undoubtedly be a great boon in the event of their coming upon the Fund; though may it not be doubtful whether any but a small proportion will do so? But is there any call for a "special recognition" or reward to those who have joined the Fund? Has not their motive been a (right) desire to benefit themselves—but not to incur any risk for the advantage of others? Am I not speaking your own views in taking this opportunity of saying that the tendency of the day to grant rewards or "special recognition"—to individuals for something they may have done—or otherwise to bring their names before the public is to be deprecated, and especially in the case of us nurses, who in consequence of the excessive attention, the *fashion*, in short—which has been opened like a sluice upon nursing matters, have by no means benefited in real nursing thoroughness or character, and are apt to become puffed up and pedantic—*not* in the right direction. I can scarcely avail myself of your kind offer to call at present, as there is really no particular occasion for discussion. And my health renders me unfit for more than a small amount of talking.

Pray believe me, faithfully yours,
(Signed) Florence Nightingale.

10 South Street, Park Lane, W., June 28, 1889.

Henry Burdett, Esq.

